# Seeking for the boundaries of de-escalation in antiplatelet therapy for acute coronary syndrome

**DOI:** 10.1093/ehjcvp/pvaf070

**Published:** 2025-09-19

**Authors:** Mattia Galli, Felice Gragnano, Marco Valgimigli

**Affiliations:** Department of Medical-Surgical Sciences and Biotechnologies, Sapienza University of Rome, Corso della Repubblica, 79, Latina 04100, Italy; Maria Cecilia Hospital, GVM Care & Research, Cotignola 48033, Italy; Department of Translational Medical Sciences, University of Campania ‘Luigi Vanvitelli’, Caserta, Italy; Division of Clinical Cardiology, A.O.R.N. ‘Sant’Anna e San Sebastiano’, Caserta, Italy; Cardiocentro Ticino Institute, Ente Ospedaliero Cantonale, Lugano, Switzerland; Department of Biomedical Sciences, University of Italian Switzerland, Lugano, Switzerland; University of Bern, Bern, Switzerland

De-escalation strategies of antiplatelet therapy have been shown to improve outcomes in patients with acute coronary syndrome (ACS).^1^ However, not all de-escalation strategies are equivalent, and some may compromise ischaemic protection compared with a standard dual antiplatelet therapy (DAPT) (*Figure 1*).^1^ Specifically, discontinuing the DAPT after 1–3 months in favour of aspirin or clopidogrel monotherapy reduces bleeding but increases ischaemic risk, whereas maintaining ticagrelor monotherapy lowers major bleeding while preserving ischaemic protection.^2,3^ Unguided de-escalation after 1–3 months, either by switching from ticagrelor or prasugrel to clopidogrel or by dose reduction (from prasugrel 10 to 5 mg o.d. or from ticagrelor 90 to 60 mg b.i.d.), has also shown encouraging results but is supported by weaker evidence. However, because these strategies are typically implemented only after 1–3 months of the standard DAPT, they fail to mitigate bleeding during the early post-ACS period, when both ischaemic and bleeding risks are highest. At variance, guided de-escalation, which is backed by evidence and endorsed in consensus documents, though not in guidelines, may be initiated immediately after ACS, offering potential benefit from the earliest weeks compared with the standard DAPT (*Figure 1*).^4^ However, its implementation is currently hindered by the limited availability of platelet function or genetic testing.^4^

**Figure 1:**
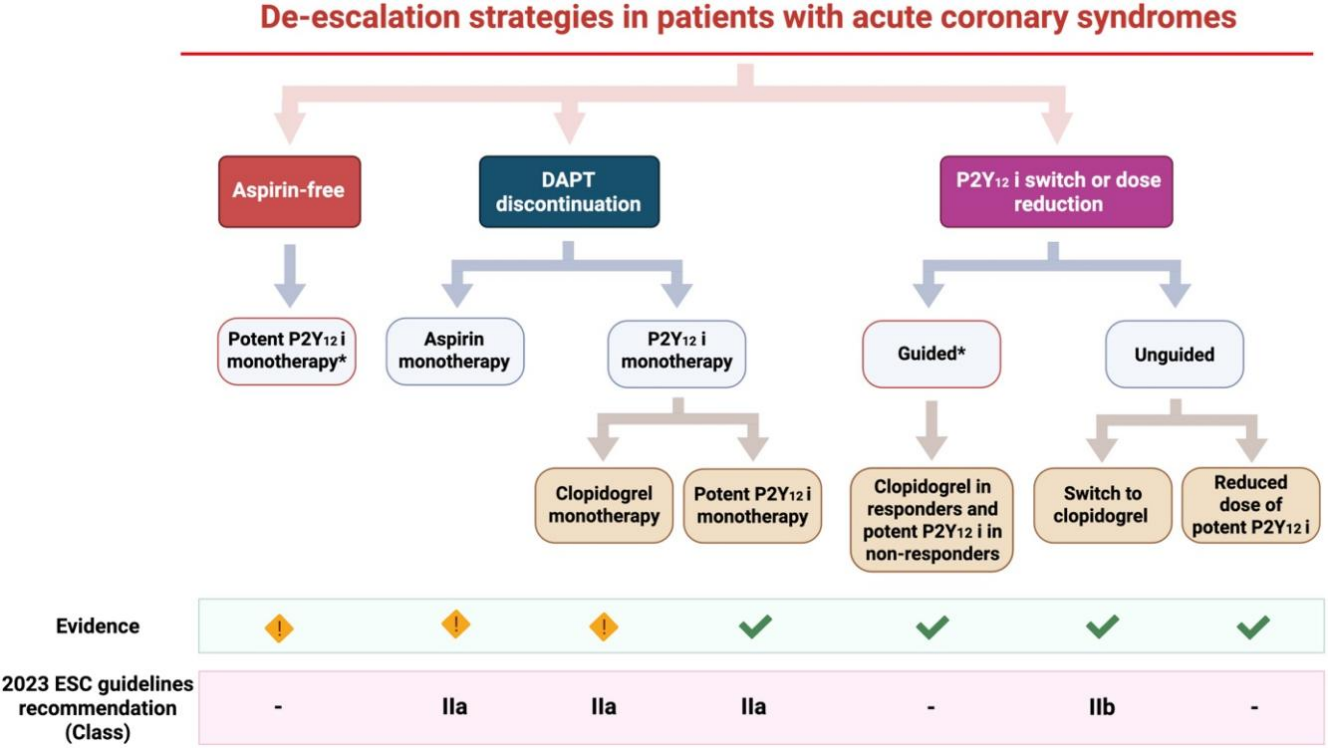
Antiplatelet de-escalation strategies in patients with acute coronary syndromes. DAPT, dual antiplatelet therapy; P2Y_12_ i, P2Y_12_ inhibitor; ESC, European Society of Cardiology. Red box and * indicate de-escalation strategies to be implemented at the time of acute coronary syndrome.

The European Society of Cardiology Congress unveiled the first large-scale trial exploring a de-escalation strategy of immediate aspirin withdrawal, with potent P2Y_12_ inhibitor monotherapy started within the first 4 days of hospitalization after successful percutaneous coronary intervention in ACS. The *Percutaneous Coronary Intervention Followed by Monotherapy Instead of Dual Antiplatelet Therapy in the Setting of Acute Coronary Syndromes* (NEO-MINDSET) trial randomized 3410 patients with ACS [62.1% with ST-segment elevation myocardial infarction (MI)] to stop treatment with aspirin and receive potent P2Y_12_ inhibitor monotherapy with prasugrel (69.6%) or ticagrelor (28%) or to receive the standard DAPT for 12 months.^5^ P2Y_12_ inhibitor monotherapy failed to demonstrate noninferiority to the DAPT for the primary endpoint of death, MI, stroke, or urgent target-vessel revascularization at 12 months.^5^ There was an absolute difference in risk of 1.47 percentage points [95% confidence interval (CI), −0.16 to 3.10], which did not meet the prespecified criteria for noninferiority (*P* = 0.10). Notably, Kaplan–Meier curves diverged within the first month after ACS, and there were numerically higher rates of secondary ischaemic events, including MI and cardiovascular death, in the monotherapy arm.^5^ At the same time, the primary composite endpoint did not significantly differ in the two study arms, which makes the interpretation of the study results not unequivocal. The incidence of major or clinically relevant nonmajor bleeding was 2.0% in the monotherapy group and 4.9% in the DAPT group [hazards ratio (HR) 0.40; 95% CI, 0.26–0.59], for a difference in risk of −2.97 percentage points (95% CI, −4.20 to −1.73). Net adverse clinical events occurred in 8.5% in the monotherapy group and 9.9% in the DAPT group (HR 0.86; 95% CI, 0.69–1.08).

These results emphasize once more that no single antiplatelet regimen fits all patients; therapy should be individualized, accounting for factors such as ischaemic and bleeding risks, as well as drug responsiveness, ethnicity, and sex, and adjusted dynamically over time.^6,7^

## Data Availability

Data availability is not applicable to this article as no new data were created or analyzed in this study.

## References

[1] Valgimigli M, Aboyans V, Angiolillo D, Atar D, Capodanno D, Halvorsen S, James S, Jüni P, Kunadian V, Landi A, Leonardi S, Mehran R, Montalescot G, Navarese EP, Niebauer J, Oliva A, Piccolo R, Price S, Storey RF, Völler H, Vranckx P, Windecker S, Fox KAA. Antithrombotic treatment strategies in patients with established coronary atherosclerotic disease. Eur Heart J Cardiovasc Pharmacother 2023;9:462–496. 10.1093/ehjcvp/pvad032 37120728 PMC12375866

[2] Valgimigli M, Hong S-J, Gragnano F, Chalkou K, Franzone A, da Costa BR, Baber U, Kim B-K, Jang Y, Chen S-L, Stone GW, Hahn J-Y, Windecker S, Gibson MC, Song YB, Ge Z, Vranckx P, Mehta S, Gwon H-C, Lopes RD, Dangas GD, McFadden EP, Angiolillo DJ, Leonardi S, Heg D, Calabrò P, Jüni P, Mehran R, Hong M-K. De-escalation to ticagrelor monotherapy versus 12 months of dual antiplatelet therapy in patients with and without acute coronary syndromes: a systematic review and individual patient-level meta-analysis of randomised trials. Lancet 2024;404:937–948. 10.1016/S0140-6736(24)01616-7 39226909

[3] Galli M, Laudani C, Occhipinti G, Spagnolo M, Gragnano F, D'Amario D, Navarese EP, Mehran R, Valgimigli M, Capodanno D, Angiolillo DJ. P2y12 inhibitor monotherapy after short DAPT in acute coronary syndrome: a systematic review and meta-analysis. Eur Heart J Cardiovasc Pharmacother 2024;10:588–598. 10.1093/ehjcvp/pvae057 39054275

[4] Angiolillo DJ, Galli M, Alexopoulos D, Aradi D, Bhatt DL, Bonello L, Capodanno D, Cavallari LH, Collet J-P, Cuisset T, Ferreiro JL, Franchi F, Geisler T, Gibson CM, Gorog DA, Gurbel PA, Jeong Y-H, Marcucci R, Siller-Matula JM, Mehran R, Neumann F-J, Pereira NL, Rizas KD, Rollini F, So DYF, Stone GW, Storey RF, Tantry US, Berg JT, Trenk D, Valgimigli M, Waksman R, Sibbing D. International consensus statement on platelet function and genetic testing in percutaneous coronary intervention: 2024 update. JACC Cardiovasc Interv 2024;17:2639–2663. 10.1016/j.jcin.2024.08.027 39603778

[5] Guimarães PO, Franken M, Tavares CAM, Antunes MO, Silveira FS, Andrade PB, Bergo RR, Joaquim RM, Tinoco de Paula JE, Nascimento BR, Pitta FG, Arruda JA, Serpa RG, Ohe LN, Mangione FM, Furtado RHM, Ferreira E, Sampaio FBA, T. do Nascimento C, Genelhu LOO, Bezerra CG, Sarmento-Leite R, Maia LN, Oliveira FRA, Wainstein MV, Dall’Orto FTC, Monfardini F, Assis SRL, Nicolau JC, Sposito AC, Lopes RD, Onuma Y, Valgimigli M, Angiolillo DJ, Serruys PWJC, Berwanger O, Bacal F, Lemos PA. Early withdrawal of aspirin after PCI in acute coronary syndromes. N Engl J Med 2025. 10.1056/NEJMoa2507980. Epub ahead of print. 40888723

[6] Galli M, Laborante R, Occhipinti G, Zito A, Spadafora L, Biondi-Zoccai G, Nerla R, Castriota F, D'Amario D, Capodanno D, Jeong Y-H, Kimura T, Mehran R, Angiolillo DJ. Impact of ethnicity on antiplatelet treatment regimens for bleeding reduction in acute coronary syndromes: a systematic review and pre-specified subgroup meta-analysis. Eur Heart J Cardiovasc Pharmacother 2024;10:158–169. 10.1093/ehjcvp/pvad085 37960983

[7] Galli M, Terracina S, Schiera E, De Corci S, Sangiorgi D, Mancone M, Frati L, Sciarretta S, Angiolillo DJ, Pulcinelli FM. Sex-related variations in platelet reactivity in presence or absence of antiplatelet therapy. Eur Heart J Cardiovasc Pharmacother 2025;11:509–517. 40366913 10.1093/ehjcvp/pvaf034 PMC12450592

